# Supplementary material for: Improving prediction of drug-target interactions based on fusing multiple features with data balancing and feature selection techniques
Source: PLoS One. 2023 Aug 3;18(8):e0288173. doi: 10.1371/journal.pone.0288173 (PMC10399861; doi:10.1371/journal.pone.0288173)
Supplement: Attachment — Submitted filename: Response to reviewers of PLOS1.docx [file pone.0288173.s001.docx]

**Dear Editor-in-Chief,**

We wish to thank you, the associate editor, and the reviewers for the comments we received on the attached paper, and also thank you for giving us the opportunity to revise the manuscript again. We hereby submit a revised version of the paper. The detailed responses to reviewers’ comments are listed as follows, where reviewers’ comments are written in bold with our reply in a Calibri font. Furthermore, due to several new simulations and experimental results comparisons, Mr. Ali Ghanbari Sorkhi, has been added as a new co-author of this paper. We hope that the modified version is acceptable and we look forward to your kind recommendations.

Best regards,

The Authors

**Reviewer Comments:**

**Response to Comments from Reviewer 1**

**This article proposes a new drug-target mutual prediction framework, which is called SRX-DTI. This method extracts the data of proteins and drugs, and proposes a new algorithm One-SVM-US to balance the data set, and then develops an algorithm called FFS-RF to predict the performance of the algorithm, and finally chooses to use XGBoost classification The device recognizes DTIs. The proposed method is compared with existing classical methods on standard datasets and defined subsets, and the results show that the proposed method has higher accuracy. However, before this manuscript is accepted, authors should address the following issues:**

We greatly appreciate the reviewer’s efforts and have made our best efforts to respond to all concerns raised by the review to the extent possible. We have made the following revisions accordingly.

**(1) In the manuscript, the author custom-divided the standard dataset into four subsets AB/CD/EF/GHI. It is recommended that the author provide the basis and method for dividing the data set in order to increase the credibility of the experiment in the article.**

Thanks for your ingenious comments and review. In the revised version, we have revised the section "Feature extraction methods" and explained the basis and method for dividing the data set. (Please see the section of Feature extraction methods technique, page 4, highlighted text.)

**(2) The paper proposes a new algorithm One-SVM-US to balance the dataset, and the pseudocode in the article is worthy of praise. It is recommended to make a more detailed description in this section to increase the credibility of the experimental comparison of the article.**

Thanks for the comments, in the revised version, we have explained the One-SVM-US algorithm more. We have also provided more details of its pseudocode. (Please see the section of Data balancing technique, pages 10 and 11, highlighted text.)

**(3) It is recommended to describe in detail the method used by the FFR-RF algorithm in Section 6.4 when selecting feature dimensions.**

Thanks for your valuable comment. In the revised version, we have added more detail for this algorithm. (Please see the section of Feature selection technique, page 11, highlighted text.)

**(4) It is recommended to introduce new data sets for comparative experiments.**

Thanks for the suggestion. We also evaluated our method on the Davis dataset and provided new comparative results. (Please see the subsection of Drug–target datasets, page 4, highlighted text, and the subsection of The influence of the data balancing techniques, pages 16 and 17, highlighted text and also, page 20, Table 14.)

**(5) The overall flowchart of the article is distorted after enlargement, and it is recommended that the author increase the clarity of the overall flowchart.**

Thanks for the comments. In revised version, we have improved the quality of flowchart.

**(6) The cited literature is insufficient, and appropriate addition of relevant literature will make the article more convincing, such as PMID 36088547, 34965582, 34456656, etc.**

Thanks for the comments. In the revised version, we have added these references. (Please see the section of Introduction, page 3, second highlighted paragraph.)

**(7) The link** [**https://github.com/Khojasteh-hb/SRX-DTI**](https://github.com/Khojasteh-hb/SRX-DTI) **in the article is not available. Authors are advised to provide readers with all the data and software code on which the conclusion of the paper depends.**

Thanks for the comments. We checked the link and it is okay. Also, the code source codes as well as the used datasets have been uploaded on GitHub.

**(8) In terms of writing technique, it is recommended to improve the condensation of sentences (especially condensed abstracts) and improve the continuity before and after certain chapters.**

Thanks for the comments, we carefully studied the paper and we completely improved the condensation of sentences and the continuity before and after certain chapters.

**Response to Comments from Reviewer 2**

**This paper proposes a new drug target interaction prediction framework called SRX-DTI. In this method, the protein sequence information and drug sequence information in the public dataset are selected to propose an algorithm called One-SVM-USD for the balanced dataset, and then the FFS-RF algorithm is developed to predict the algorithm performance, and XGBoost is selected for classification after experimental comparison. Finally, the overall performance is verified experimentally, and the results show that the framework performs well. However, before the manuscript is accepted, authors are advised to make improvements in the following areas:**

We greatly appreciate the reviewer’s efforts to carefully review the paper and the valuable suggestions offered. We have made the following revisions accordingly.

**(1) It is suggested that the authors provide the basis and method for dividing the four subsets of AB/CD/EF/GHI.**

Thanks for your ingenious comments and review. In the revised version, we have revised the section "Feature extraction methods" and explained the basis and method for dividing the data set. (Please see the section of Feature extraction methods technique, page 4, highlighted text.)

**(2) This paper proposes a new algorithm One-SVM-US to balance the dataset, suggesting that the authors describe its innovation points in detail.**

Thanks for the comments, in the revised version, we have explained the One-SVM-US algorithm more. We have also provided more details of its pseudocode. (Please see the section of Data balancing technique, page 10 and 11, highlighted text.)

**(3) The 08 data set is used in the article, and it is recommended that the author introduce a new data set.**

Thanks for the suggestion. We also evaluated our method on the Davis dataset and provided new comparative results. (Please see the subsection of Drug–target datasets, page 4, highlighted text, and the subsection of The influence of the data balancing techniques, pages 16 and 17, highlighted text and also, page 20, Table 14.)

**(4) Authors are advised to improve the flowchart framework and improve its clarity.**

Thanks for the comments. In revised version, we have improved the quality of flowchart.

**(5) The link** [**https://github.com/Khojasteh-hb/SRX-DTI**](https://github.com/Khojasteh-hb/SRX-DTI) **in the article is not available.
It is recommended that authors provide more accurate and realistic code and data.**

Thanks for the comments. We checked the link and it is okay. Also, the code source codes as well as the used datasets have been uploaded on GitHub.

**(6) In terms of writing techniques, it is recommended to improve the grammar and improve the logic of the chapters.**

Thanks for the comments, we carefully studied the paper and we completely improved the grammar and the continuity before and after certain chapters.

**(7) It is recommended to increase the diversity of the table.**

Thanks for the suggestion. We increased the diversity of the table and added tables 13 and 14, which report the AUROC values under the 5-Fold cross-validation.

**Response to Comments from Reviewer 3**

**The author reported a computational method, called SRX-DTI. They proposed FFS-RF algorithm and present One-SVM-US technique. The results show superior performance in predicting DTIs. Besides, I still have some questions need to be solved.**

We greatly appreciate the reviewer’s efforts to carefully review the paper and the valuable suggestions offered. We have made the following revisions accordingly.

**1. In Abstract, the representation needs to be revised. I advised the author to read some related articles, to learn how the other authors represent. Generally, the logic of the article is not clear enough, need to be improved.**

Thanks for the comments. We studied related articles and we have improved the abstract.

**2. In Introduction, I know the topic of this paper is drug-target interaction. But the authors talk about Protein-protein interaction, It’s no problem. But I did not know the relationship between this paragraph. The logical needs to be sorted out.**

Thanks for the comments, we carefully reviewed the paper and we completely improved the condensation of sentences and the relationship between those paragraphs.

**3. In feature extraction section, the author proposed nine protein features, and construct for group, I want to know why these features only use four subsets? How about five groups? Or six? It needs to be clear expression or further talk about this reason.**

Thanks for your ingenious comments and review. In the revised version, we have revised the section "Feature extraction methods" and explained the basis and method for dividing the data set. (Please see the section of Feature extraction methods technique, page 4, highlighted text.)

**4. I want to know One-SVM-US. What the full name is?**

Thanks for your comment. The full name of One-SVM-US is UnderSampling by One-class Support Vector Machine. In the revised version, I have added the full name in the first mention of this name. (Please see the section of Introduction, page 3, third highlighted paragraph.)

**5. The picture in the text is too blurred. The dpi can be set more than 300.**

Thanks for the comments. In revised version, we have improved the quality of figures.

**6. if author have further extensive experiment.**

Thanks for the suggestion. We also evaluated our method on the Davis dataset and provided new comparative results. (Please see the subsection of Drug–target datasets, page 4, highlighted text, and the subsection of The influence of the data balancing techniques, pages 16 and 17, highlighted text and also, page 20, Table 14.)

**7. so what’s the conclusion of this whole paper? And which the limitation of this work. Please show me.**

Thanks for your ingenious comments and review. In the revised version, we have revised the conclusion section and mentioned the limitation of this work.

**In conclusion, I think this manuscript need to be large revised (mainly about the expression). And improve the English expression.**

Thanks for your comments. In the revised version, we have completely improved the English expression and grammar.
